# Incidence of notified Lyme borreliosis in Germany, 2013–2017

**DOI:** 10.1038/s41598-018-33136-0

**Published:** 2018-10-08

**Authors:** Julia Enkelmann, Merle Böhmer, Volker Fingerle, Claudia Siffczyk, Dirk Werber, Martina Littmann, Sophie-Susann Merbecks, Carina Helmeke, Sabine Schroeder, Stefan Hell, Uwe Schlotthauer, Florian Burckhardt, Klaus Stark, Anika Schielke, Hendrik Wilking

**Affiliations:** 1 Postgraduate Training for Applied Epidemiology, Robert Koch Institute, Berlin, Germany; 2 Department of Infectious Disease Epidemiology, Robert Koch Institute, Berlin, Germany; 3 Department of Public Health Microbiology & Infectious Disease Epidemiology and National Reference Centre for Borrelia, Bavarian Health and Food Safety Authority, Oberschleissheim, Germany; 4 Brandenburg State Office of Occupational Safety, Consumer Protection and Health, Potsdam, Germany; 5 State Office for Health and Social Affairs, Berlin, Germany; 6 State Office for Health and Social Affairs, Rostock, Mecklenburg-Western Pomerania Germany; 7 State Health Authority Saxony, Chemnitz, Germany; 8 State Agency for Consumer Protection of Saxony-Anhalt, Halle (Saale), Germany; 9 Thuringian State Authority for Consumer Protection, Bad Langensalza, Germany; 10 State Authority of Saarland for Social Affairs, Health, Women and Family, Berlin, Germany; 11 Institute of Medical Microbiology and Hygiene, Saarland University, Homburg/Saar, Germany; 12 Federal State Agency for Consumer & Health Protection, Rhineland- Palatinate, Germany

## Abstract

Lyme borreliosis (LB) is the most commonly reported tick-borne disease in Germany. In 9/16 states, notification of erythema migrans (EM), acute neuroborreliosis (NB) and Lyme arthritis (LA) is mandatory. We describe incidence measures, time trends, geographical distribution and frequencies of manifestations to better understand LB epidemiology and target prevention measures. We used cases notified in the 9 states and confirmed by local health offices, 2013–2017, to calculate incidences by time, place and person. Altogether, we observed 56,446 cases. Disease onset peaked yearly in July. Incidence ranged from 26/100,000 (2015) to 41/100,000 (2013) with mean annual incidences 2013–2017 on district level between 0.5/100,000 and 138/100,000. Median age was 54 years with peaks in boys (5–9 years, mean incidence 36/100,000) and women (50–69 years, mean incidence 57/100,000). 95% experienced EM only, 2.7% NB and 2.1% LA. 54% were female, but more men had NB (56%) and LA (53%, p < 0.001). Hospitalisation was recorded for 10% of LA and 71% of NB cases. LB remains an important public health concern in Germany with marked regional variation. To facilitate early diagnosis and treatment, health authorities should raise awareness among physicians and promote prevention strategies among the general population: tick-bite-protection, prompt tick removal and medical consultation.

## Introduction

Lyme borreliosis (LB) is the most frequently reported tick-borne disease in Germany. It is caused by spirochetes of the *Borrelia burgdorferi* sensu lato complex and is transmitted to humans by bites of infected *Ixodes* species ticks^1,2^. LB is a multi-system disease. The most common clinical manifestation is erythema migrans (EM)^3^. If the bacteria disseminate, more severe forms like neuroborreliosis (NB), Lyme arthritis (LA) or less frequently Lyme carditis can occur^4,5^. In Germany, an effective vaccine is currently not available and prevention strategies concentrate on raising awareness, avoiding tick-bites, checking for ticks, prompt removal of attached ticks and early medical consultation if symptoms occur^6^. If treated early with appropriate antibiotics the prognosis is good^7^.

Representative seroprevalence studies have demonstrated that infections with *Borrelia burgdorferi* occur all over Germany^8,9^. However, seroconversions may be asymptomatic and antibodies may persist for many years even if treated appropriately^10^. A prospective population-based study conducted 1996 in a city in the south of Germany found LB incidences of 111/100,000 population^11^. Analysis of medical claims data from 2007–2008 of a medical insurance provider with more than 6 million members has estimated annual incidences of 261/100,000 population amounting to almost 214,000 annual cases of LB if extrapolated to Germany with direct medical costs of more than 74 million Euro per year^12,13^. Notification data restricted to East German states 2009–2012 reported incidences ranging from 19.5/100,000 population in 2009 to 34.9/100,000 population in 2012^14^.

Since 2013 mandatory notification for the three most common LB manifestations (EM, acute NB and LA) is implemented in 9 of 16 German states covering 42% of the total German population. As knowledge on incidence, frequency of clinical manifestations and geographical distribution is still incomplete and public interest is high, we describe incidence measures of notified LB and present time trends, geographical distribution, frequency of clinical manifestations and associations with demographic characteristics based on available surveillance data to inform stakeholders and target prevention measures.

## Results

### Time trends

**Figure 1:**
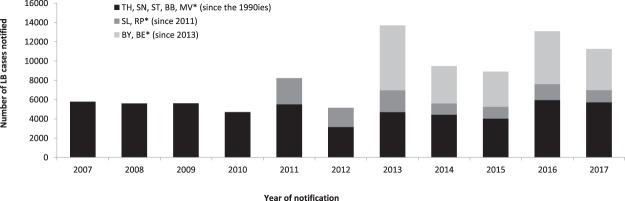
Number of notified LB cases by year of notification and states grouped according to year of implementation or change in LB surveillance. Data from 2007 to 2012 was included to allow comparison. The following changes were made in the implementation: In 2009 LA was included as notifiable manifestation of LB in all notifying states, in 2011 notification was introduced in RP (July) and SL (August), in 2013 notification was introduced in BY (data included since 1^st^ of April 2013) and extended in BE (data included in Figure 1 since 1^st^ January 2013). *Thuringia (TH), Saxony (SN), Saxony-Anhalt (ST), Brandenburg (BB), Mecklenburg-Western Pomerania (MV), Rhineland-Palatinate (RP), Saarland (SL), Bavaria (BY), Berlin (BE).

Between 2013 and 2017, 56,446 cases of LB (annual mean 11,289; range 8,902–13,708) were reported in the 9 states with mandatory notification, corresponding to a mean annual incidence 2013–2017 of 33/100,000 population (range: 26/100,000 (95% confidence interval (CI): 25.6–26.7) in 2015 to 41/100,000 (95% CI: 40.2–41.6) in 2013). There was no obvious increasing or decreasing trend over time (Fig. 1). The highest number of cases was notified in 2013, especially in states with new implementation of surveillance. In 2014 and 2015 numbers were lower and increased again in 2016.

### Marked seasonality with peak in July

**Figure 2:**
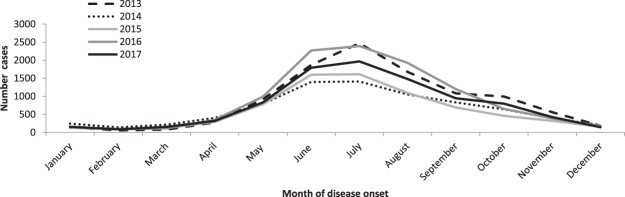
Number of LB cases by month of disease onset and year of notification in 9 German states, 2013–2017.

Information on month of disease onset was available for 80% of cases. LB displayed a marked seasonal pattern (Figure 2). Fifty-seven percent of cases reported disease onset between June and August, peaking in July every year. Seasonality was stable from 2013 to 2017.

**Figure 3:**
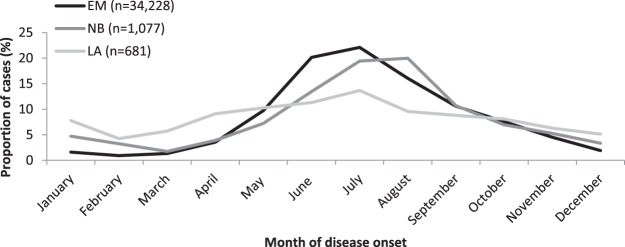
Proportion of LB cases by month of disease onset and clinical manifestation in 9 German states, 2013–2017.

Seasonality differed depending on clinical manifestation (Figure 3). EM cases peaked in July (22%), NB cases in August (20%) and LA cases were more evenly distributed throughout the year with only 35% reporting disease onset between June and August.

### Regional variation

LB occurred in all areas under surveillance but there was marked regional variation. Despite annual fluctuations in LB incidence overall, the same regions displayed higher LB incidences during the different years of the study period.

Based on mean annual incidence 2013–2017, we observed five regions of particularly high incidence.

**Figure 4:**
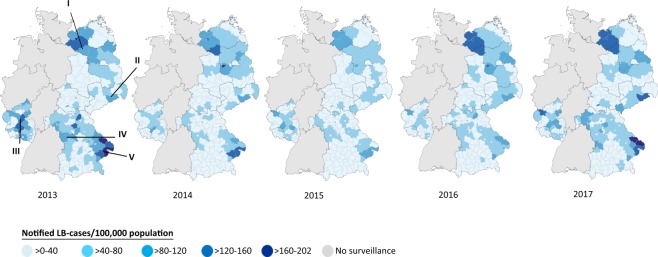
Notified LB incidence by district of residence (n = 56,011). Based on equal distance between lowest and highest recorded incidence, we formed 5 incidence categories. 435 cases with tick exposure in a foreign country were excluded. Among the remaining 33,153 cases with information on place of tick exposure in Germany, district of exposure corresponded with district of residence in 90.6%, 4.9% reported exposure in another district in the same state and 4.5% in another state in Germany.

In Figure 4 they are depicted from 2013–2017: (I) West-Mecklenburg including the city of Schwerin and North-Brandenburg (especially districts Prignitz, Brandenburg an der Havel, Barnim and Potsdam-Mittelmark), (II) the Erz mountains, (III) Hunsrueck/Eifel region (especially districts Vulkaneifel and Birkenfeld), (IV) Fraenkische Alb/Steigerwald (especially districts Ansbach and Donau Ries) and (V) the Bavarian forest. In the Bavarian forest (V) and Hunsrueck/Eifel (III) incidence was highest in 2013, in the Erz mountains (II) in 2016 and in West-Mecklenburg/Prignitz (I) 2016/2017.

The highest annual incidence on district level was 202/100,000 population in district Regen, recorded in 2013.

The mean annual incidence 2013–2017 on district level was highest in the city of Schwerin (MV, 138/100,000/year) and lowest in district Kaufbeuren (BY, 0.5/100,000/year).

### Demographic characteristics and clinical information

#### Bimodal age distribution

**Figure 5:**
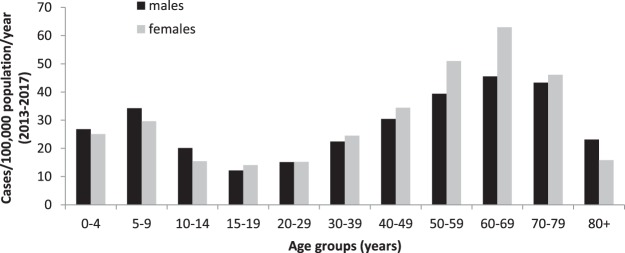
LB incidence by age group and sex in 9 German states, 2013–2017.

Median age was 54 years (interquartile range 39–65 years). Of all notified LB cases 54% were female. We observed bimodal age distribution with incidence-peaks in boys aged 5–9 years (corresponding to a mean incidence 34/100,000 population/year, 2013–2017) and women aged 50–69 years (corresponding to a mean incidence of 57/100,000 population/year) (Figure 5).

#### Predominant clinical manifestation erythema migrans

Detailed information on clinical manifestations was available for 99% of cases. Thereof, 95% had EM only and 4.8% suffered from more severe forms: 2.7% had NB and 2.1% LA (Table 1).

**Table 1:** Clinical manifestations and hospitalisation of LB-cases notified in 9 German states, 2013–2017.

Manifestation	Number of cases	In %	Number of hospitalisation/all cases with available information	In %
Erythema migrans only	**53,177**	**95.3** ^a^	919/43,419	2.0
Acute neuroborreliosis, total	**1,481**	**2.7** ^a^	972/1,360	71.5
Thereof ^b^				
Cranial nerve palsy	668	45.1^c^	560/628	89.2
Radiculoneuritis	620	41.9^c^	316/568	55.6
Meningitis	287	19.4^c^	246/271	90.8
Neuroborreliosis not differentiated^b^	121	8.2^c^	37/95	39.0
Lyme arthritis	**1,182**	**2.1** ^a^	97/1,017	9.5
Cases with information	55,812^d^		45,711^e^	

^a^Percent of all cases; ^b^entry of multiple NB manifestations possible, ^c^percentage of all NB cases, ^d^28 cases NB plus LA, ^e^25 cases NB plus LA.

Proportions of cases with NB decreased from 3.2% in 2013 to 2.0% in 2017. The most commonly reported manifestation of NB shifted from radiculoneuritis (most common form 2013–2015) to cranial nerve palsy in 2016. Proportion of cases with LA decreased from 3.3% in 2014 to 1.5% in 2017.

4.4% of all cases with available information had a documented hospital admission (range 2013–2017: 3.9–5.3%). Depending on clinical manifestation this varied between 2.1% if only EM was present, 10% in cases with LA and 71% in patients with NB.

While 55% of EM cases were observed among females, more males reported extracutaneous manifestations: 56% of NB cases (Incidence rate ratio (IRR) 1.3, 95% CI 1.2–1.4) and 53% of LA cases (IRR 1.2, 95% CI 1.1–1.3) affected males.

**Figure 6:**
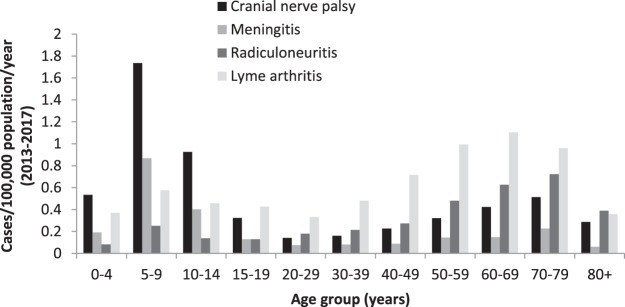
Incidence of LA and different manifestations of NB by age group in 9 German states, 2013–2017.

We observed significant differences in clinical manifestations depending on age and sex (Table 2 and Figure 6). Incidence of cranial nerve palsy was especially high in children 5–9-years with IRR of 12.8 (95% CI: 8.4–19.6) as well as meningitis with IRR of 14.1 (95% CI: 7.6–26.3) when compared to people aged 20–29 years (reference age group, Table 2). LA incidences followed a bimodal age distribution with highest incidences among adults aged 60–69 years (IRR compared to people aged 20–29 years: 3.5, 95% CI: 2.7–4.7).

**Table 2:** Incidence rate ratios (IRR) for clinical manifestations depending on sex and age group, LB cases notified in 9 German states, 2013–2017.

	Radiculoneuritis		Cranial nerve palsy		Meningitis		Lyme arthritis	
	IRR	95% CI	IRR	95% CI	IRR	95% CI	IRR	95% CI
Male	1.2	1.1–1.4	1.5	1.3–1.7	1.2	0.9–1.5	1.2	1.1–1.3
Age 5–9 years^a^	1.4	0.8–2.5	12.8	8.4–19.6	14.1	7.6–26.3	1.8	1.2–2.6
Age 50–59 years^a^	2.7	1.9–3.9	2.4	1.5–3.7	2.4	1.2–4.6	3.1	2.4–4.1
Age 60–69 years^a^	3.5	2.4–5.1	3.2	2.1–5–0	2.5	1.3–4.8	3.5	2.7–4.7

^a^Reference age group: 20–29 years.

#### 71% recalled a tick-bite

71% of cases with available information (n = 45,489) recalled a tick-bite prior to illness. Recall of tick-bite varied depending on clinical manifestation. People with EM recalled a tick-bite most frequently (72%), followed by people with NB (50%) and people with LA (41%). We did not detect differences in recall between males and females and only small differences between children and adults (68% versus 71%, p < 0.01).

Among cases with available information, district of tick exposure differed from district of residence in 11% of cases: 4.8% reported exposure in another district in the same state, 4.4% in another state and 1.3% abroad.

#### Genospecies

Detailed information on causative genospecies was only available for 299 cases (0.5%). The most commonly demonstrated genospecies was *B*. *burgdorferi* sensu stricto (186/299; 62%), followed by *B*. *afzelii* (86/299; 29%), *B*. *garinii* (16/299; 5.4%), *B*. *bavariensis* (7/299; 2.3%) and *B*. *spielmanii* (4/299; 1.2%).

## Discussion

This report provides information on the epidemiology of notified LB for 9 out of 16 German states. Three states implemented LB surveillance between 2011 and 2013 and are included for the first time. We observed annual fluctuations in reported numbers of LB cases but no clear increasing or decreasing trend. Incidence of LB was especially high in 2013 and 2016 which might be explained by favourable weather conditions for recreational activities and differences in tick abundance although new implementation of notification in 2013 might have contributed to the peak in the form of a surveillance artefact. Cases of tick-borne encephalitis (TBE) – the other important tick-borne infection in Germany – showed a similar pattern as LB for 2013–2016, but not for 2017^15^. In 2017 a higher number of TBE was reported compared to 2016 while the number of LB cases in most states slightly decreased. However, TBE differs from LB in many aspects: it is a viral vaccine-preventable disease with focal distribution primarily occurring in the south of Germany and large parts of affected regions are not under LB surveillance.

Most patients reported onset of symptoms in the summer months as can be expected with the seasonal pattern of the tick’s host seeking behavior. Seasonality remained stable 2013–2017. As disseminated forms may only become clinically manifest up to weeks and month after infection, the observed differences in disease onset depending on manifestation are not surprising. Most NB-cases had disease onset in July and August with decline in September and only few cases during autumn, winter and early spring. The pattern was similar to that of EM, just postponed by one month suggesting that the majority of NB cases develop manifest disease in a well-described time span after their tick-bite. In contrast LA displayed a more even distribution indicating a more variable and longer incubation period.

All regions under surveillance reported cases of LB. Despite fluctuations in intensity, some regions showed markedly higher incidences throughout 2013–2017. The regions in West Mecklenburg, North Brandenburg and the Erz mountains in Saxony had already been identified as high incidence areas in LB surveillance restricted to the Eastern German states 2009–2012^14^. Although some regions consistently showed low reporting incidences, no region can be declared free of LB. Due to existing differences in notification system (notifying body, anonymous versus by name) and available resources to operate it and raise awareness, the relation between reported and unreported cases might have been different for different districts and states.

In the majority of cases tick exposure occurred in the district of residence. This seems to also be true for TBE, where tick exposure occurred in the district of residence in 87% of cases^16^. Additionally, a citizen science research program on tick-bites recorded by the public in the Netherlands suggests tick-bites might frequently be acquired close to home; after bites in forests, gardens were the second most common mentioned places (31%) where tick-bites occurred^17^.

The populations in which most cases occurred in the 9 states remained similar to those outlined in previous reports of German LB surveillance data as well as results of a prospective population based study in South Germany 1996^11,14^. Bimodal age distribution with peaks in boys aged 5–9 years and females 50–69 years is consistent with representative German serosurveys^8,9^ and has been observed in previous studies and in other countries^14,18^. In the US the second peak occurred at a slightly younger age in adults aged 50–54 years^19^. A Dutch research project on tick-bites based on reporting by the public and the passive tick surveillance system in Quebec, Canada, based on ticks collected from human patients in participating hospitals, found children below 10–15 years and adults between 50 and 69 years were bitten most commonly, suggesting disparities in tick exposure as possible explanation^17,20^. This is supported by a similar age distribution observed in TBE^16^ and may be due to differences in leisure behaviour. Representative German serosurveys support the bimodal age distribution, therefore not in keeping with different health seeking behaviour as sole underlying reason. Another hypothesis would be that the rate of seroconversion and clinical manifestation are higher in specific age groups, but to our knowledge there are no studies that support this.

The male female ratio in adults is discordant with representative German serosurveys which showed significantly higher seroprevalence for *B*. *burgdorferi* s.l. in adult males (13% versus 5.8% in females, p < 0.01)^9^. Different manifestation rates and health seeking behavior have been proposed by Wilking *et al*. as possible reasons^14^. In TBE higher incidences were observed in males across all age groups (exception: age 40–59 years in 2016)^16^.

The predominant reported clinical manifestation was EM only, with similar proportions as the 94% recorded in French sentinel data^18^. Proportions of disseminated forms were higher in prospective population based studies in Sweden (both NB 16% and LA 7%) and the south of Germany (LA 5%, NB 2.9%) and surveillance data from the US which also showed much higher proportions of LA (LA 27.5%, NB 12.5%), but cases in North America are predominantly caused by *B*. *burgdorferi* sensu stricto, which is known to have a predilection to cause arthritis^5^.

Acute NB was the most common disseminated form and cranial nerve palsy the most frequently observed manifestation of NB. This is concordant with a retrospective analysis of hospitalised NB cases in Hanover where cranial nerve palsy most frequently led to hospitalisation in the cohort of NB patients and surveillance data from the US^19,21^ but is in contrast to earlier German surveillance data where radiculoneuritis was the most commonly recorded manifestation of NB^14^.

Overall decreasing trends in disseminated manifestations could be an indication of increased awareness of LB in the population and among physicians leading to earlier diagnosis and initiation of treatment but more data from clinical epidemiological studies is needed to confirm this. Proportions of disseminated manifestations may be lower than expected from other studies as case definitions for LA and NB were complex and very specific. In this analysis, 170 cases with neurological symptoms and 261 cases with arthralgia were not considered as NB or LA, as it couldn’t be determined that all criteria required to fulfill the case definition for NB or LA were met.

Our observation that females were more frequently affected by EM whereas more males presented with extracutaneous forms is in keeping with a Slovenian study looking at gender disparities between cutaneous and non-cutaneous manifestations of LB. It found an even more pronounced male predominance among NB (61%) and LA (75%) and the female predominance of skin manifestations to include the late form acrodermatitis chronica atrophicans (ACA), which is not under surveillance in Germany^22^. Our main hypothesis is that German men might be less likely to seek medical attention for EM. In that case they would not be captured in the notification system resulting in a higher degree of underreporting in males. Additionally, they would miss opportunities to be treated early and consequently become at higher risk for disseminated more serious forms of LB. The finding that ACA- another late manifestation of LB- was more commonly observed in females in the Slovenian study would speak against this, but the authors hypothesised that females in Slovenia are more likely to seek medical attention for any skin lesion. We assume health seeking behaviour is less likely to differ between males and females in case of NB or LA.

Biological hypotheses include lower infection rate with genospecies that have a propensity to affect the brain or joints and different likelihood of spirochetemia leading to disseminated forms according to gender, but sufficient evidence is lacking^22^. However, gender differences in childhood NB have been described in a Norwegian study; facial nerve palsy was more commonly observed in girls and headache and/or neck stiffness as only symptom was more commonly observed in boys. A higher level of cerebrospinal fluid inflammation was found in boys independent of manifestation^23^.

Higher incidences of NB in children have been demonstrated in previous studies with the two most common manifestations being cranial nerve palsy followed by meningitis^11,24,25^. It has been proposed this could be attributable to the association between NB and tick-bites in the neck and head region, which are more commonly observed in children^11,25^.

When estimating the burden of LB for Germany as a whole, additional data sources need to be taken into account as solely relying on surveillance data could lead to under-prioritization of the problem. As with other notifiable diseases undiagnosed (cases where medical attention was not sought or where a diagnosis was not made) and unreported cases are not captured in the surveillance data. Although considerable underreporting has to be assumed the extent of underreporting in Germany is currently unknown. CDC assumes number of LB cases to be 10 fold higher than depicted by surveillance data^26^. Although misdiagnosis also occurs, differences in German LB incidence estimates based on medical claims data (260/100,000)^13^ and notification data (mean annual incidence 33/100,000) suggest the degree of underreporting might be similar in Germany.

Surveillance data can still be useful in indicating trends over time, geographical distribution and identification of particularly affected groups within the population to help prioritizing public health action and resources. However, running a surveillance system poses a substantial burden on physicians, laboratories and public health authorities. Therefore, in a country with high incidence and ubiquitous distribution like Germany consideration should be given to possible alternatives which might be more efficient to obtain estimates; especially since in the absence of human to human transmission public health authorities do not need to implement infection control measures for individual cases.

For example, in the US LB is nationally notifiable since 1991 and health jurisdictions receive notifications from laboratories and physicians. Laboratory reports are further investigated to allow classification and to determine if the clinical picture satisfies the case definition. Some states with a high burden of LB have recently started to investigate a pre-defined subset of laboratory reports of *Borrelia burgdorferi* intensively and extrapolate results to the other laboratory notifications to obtain an estimate of true numbers^19^. The rationale is that continuous surveillance is costly, does not result in new information and is taking up limited resources that might be spent strengthening prevention measures^19^. Other alternatives could be sentinels like implemented in France, solely laboratory reporting like implemented in Denmark or the periodical conduction of prospective studies. Ideally an alternative structure should also be able to monitor regional variations.

Effective prevention of LB has proven challenging. In terms of landscaping measures, application of acaricides to residential yards decreased tick abundance but did not significantly reduce the risk of tick exposure or incidence of Lyme disease^27^. In a population-based study in Connecticut fencing in the yard was the only identified protective landscaping measure^28^. Adherence to existing behavioral recommendations has been poor even in high-incidence areas^29^.

In conclusion LB remains an important public health concern in Germany. It is prevalent in all regions under surveillance and occurs in all age groups. In the absence of a vaccine, health authorities should continue to promote prevention strategies including tick-bite prevention and prompt tick removal among the general population. To facilitate early diagnosis and treatment which can prevent more severe courses of disease the population should also be informed about clinical manifestations especially erythema migrans to be able to seek medical consultation early. Our data might help to adjust communication strategies to identified risk groups: especially affected age groups and people living in regions identified as having high-incidence of LB. Furthermore, our data suggests that health seeking behaviour may differ according to gender, but this needs to be confirmed with further studies. Awareness of LB occurrence in the region can also assist local physicians with clinical considerations. Moving forward new prevention strategies, especially the availability of an effective vaccine would be beneficial.

## Methods

LB surveillance covered 42% of the total German population and included the three most common clinical manifestations: clinically diagnosed EM, laboratory confirmed LA and laboratory confirmed acute NB presenting as cranial nerve palsy, radiculoneuritis or meningitis. In 8 of 9 states physicians and in 5 of 9 states laboratories were obliged to report such cases to the local public health authorities. 4 of 9 states reported by name, the other 5 states reported anonymously.

To allow comparison with previous analyses of German LB surveillance data, we applied the same case definition: Laboratory confirmation of radiculoneuritis and meningitis required lymphocytic pleocytosis in cerebrospinal fluid (CSF) plus one of the following: (i.) culture of pathogen (CSF), (ii.) detection of pathogen DNA by PCR (CSF) or (iii.) demonstration of intrathecal antibody production (CSF, serum). Cranial nerve palsy required confirmation by (i.) culture of pathogen (CSF), (ii.) detection of pathogen DNA by PCR (CSF), (iii.) demonstration of intrathecal antibody production (CSF, serum) or in cases aged 18 years and below (iv.) IgG antibodies (serum). LA was defined as mono- or oligoarthritis for which other etiologies were excluded with laboratory confirmation by one of the following (i.) detection of IgG antibodies (serum), (ii.) culture of pathogen (joint) or (iii.) detection of pathogen by PCR (joint).

The six neighboring Eastern states BE, BB, MV, SN, ST and TH first established LB notification in the 1990ies. In 2011 two states in Western Germany (RP and SL) and in 2013 BY in South Germany implemented mandatory surveillance. BE has extended existing notification for physicians to also include laboratories in 2013.

Surveillance data was captured by district of residence. Local public health offices transmitted notifications fulfilling the case definition to the federal public health offices in anonymised form. From there they were forwarded to the national public health institute (Robert Koch Institute, RKI).

We included all LB cases notified between 01.01.2013 and 31.12.2017 as of 01.01.2018 for which local health offices confirmed that the case definition was met. Data from BY was included from 01.04.2013. Cases were classified as NB if neuroborreliosis in general or clinical pictures of radiculoneuritis, meningitis or cranial nerve palsy were reported or transmitted in the comment field. Cases were classified as LA if arthritis was reported as symptom or in the comment field. In cases with concurrent EM and NB or LA it was required that sufficient evidence of laboratory confirmation for the disseminated form was provided.

We used population figures from the Federal Office of Statistics to calculate LB incidences by time (annual incidences and mean annual incidences 2013–2017), place and person, IRRs and 95% confidence intervals (CI). Stata 14.1 (StataCorp. 2015. *Stata Statistical Software*: *Release 14*. College Station, TX, StataCorp LP) and MS Excel 2010 were used to carry out descriptive statistic and regression analysis and RegioGraph Analyse (GfK GeoMarketing GmbH, Bruchsal, Germany) to generate incidence maps on district level.

## Data Availability

This data is available in aggregated form on an online platform of the Robert Koch Institute for download (https://survstat.rki.de). Individual-based data is not publicly available due to data protection restrictions inside the legal framework for surveillance.
