# Author Correction: Incidence of notified Lyme borreliosis in Germany, 2013–2017

**DOI:** 10.1038/s41598-021-92398-3

**Published:** 2021-06-21

**Authors:** Julia Enkelmann, Merle Böhmer, Volker Fingerle, Claudia Siffczyk, Dirk Werber, Martina Littmann, Sophie-Susann Merbecks, Carina Helmeke, Sabine Schroeder, Stefan Hell, Uwe Schlotthauer, Florian Burckhardt, Klaus Stark, Anika Schielke, Hendrik Wilking

**Affiliations:** 1 Postgraduate Training for Applied Epidemiology, Robert Koch Institute, Berlin, Germany; 2 Department of Infectious Disease Epidemiology, Robert Koch Institute, Berlin, Germany; 3 Department of Public Health Microbiology & Infectious Disease Epidemiology and National Reference Centre for Borrelia, Bavarian Health and Food Safety Authority, Oberschleissheim, Germany; 4 Brandenburg State Office of Occupational Safety, Consumer Protection and Health, Potsdam, Germany; 5 State Office for Health and Social Affairs, Berlin, Germany; 6 State Office for Health and Social Affairs, Rostock, Mecklenburg-Western Pomerania Germany; 7 State Health Authority Saxony, Chemnitz, Germany; 8 State Agency for Consumer Protection of Saxony-Anhalt, Halle (Saale), Germany; 9 Thuringian State Authority for Consumer Protection, Bad Langensalza, Germany; 10 State Authority of Saarland for Social Affairs, Health, Women and Family, Berlin, Germany; 11 Institute of Medical Microbiology and Hygiene, Saarland University, Homburg/Saar, Germany; 12 Federal State Agency for Consumer & Health Protection, Rhineland-Palatinate, Germany

Correction to: *Scientific Reports* 10.1038/s41598-018-33136-0, published online 08 October 2018

The original version of this Article contained an error in Figure 4, where the years 2016 and 2017 have been reversed. In addition, data in the label for the first category was incorrect:

“> 0–40”.

now reads:

“≤ 40”.

The original Figure 4 and accompanying legend appear below.

**Figure 4:**
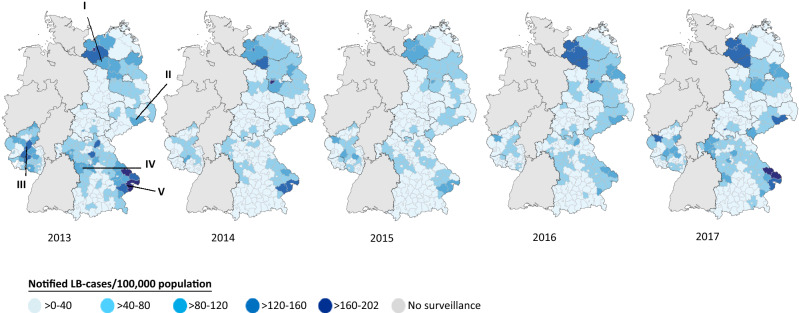
Notified LB incidence by district of residence (n = 56,011). Based on equal distance between lowest and highest recorded incidence, we formed 5 incidence categories. 435 cases with tick exposure in a foreign country were excluded. Among the remaining 33,153 cases with information on place of tick exposure in Germany, district of exposure corresponded with district of residence in 90.6%, 4.9% reported exposure in another district in the same state and 4.5% in another state in Germany.

The original Article has been corrected.

